# Assessment of heavy metal pollution in Vistula river (Poland) sediments by using magnetic methods

**DOI:** 10.1007/s11356-020-08608-4

**Published:** 2020-04-17

**Authors:** Iga Szczepaniak-Wnuk, Beata Górka-Kostrubiec, Sylwia Dytłow, Piotr Szwarczewski, Piotr Kwapuliński, Jakub Karasiński

**Affiliations:** 1 Institute of Geophysics, Polish Academy of Sciences, Ks. Janusza 64, 01-452 Warsaw, Poland; 2 Faculty of Geography and Regional Studies, University of Warsaw, Krakowskie Przedmieście 30, 00-927 Warsaw, Poland; 3 Institute of Materials Science, University of Silesia, 75 Pułku Piechoty 1A, Chorzow, Poland; 4 Faculty of Chemistry, Biological and Chemical Research Centre, University of Warsaw, Żwirki i Wigury 101, 02-089 Warsaw, Poland

**Keywords:** Magnetic methods, River sediments, Heavy metals, Magnetic spherules

## Abstract

The present study evaluated the level of heavy metal (HM) pollution in Vistula river sediments in a highly urbanized Warsaw agglomeration (Poland). Magnetometry was used to assess the pollution level by measuring the fine fractions (0.071 mm and < 0.071 mm) of sediments collected from the surface layer of the riverbank. The magnetic methods (e.g., mass magnetic susceptibility *χ*, temperature-dependence magnetic susceptibility, and hysteresis loop parameters) were supplemented by microscopy observations and chemical element analyses. The results showed the local impact of Warsaw’s activity on the level of HM pollution, indicated by the maximum concentrations of magnetic particles and HM in the city center. The sediment fraction < 0.071 mm was dominated by magnetite and by a large amount of spherical-shaped anthropogenic magnetic particles. The pollution from the center of Warsaw was transported down-river over a relatively short distance of approximately 11 km. There was a gradual decrease in the concentrations of magnetic particles and HM in areas located to the north of the city center (down-river); furthermore, *χ* and concentrations of HM did not decrease to the values observed for the area to the south of Warsaw (up-river). The study showed two possible sources of sediment pollution: traffic-related and heat and power plant emissions. The influence of an additional source of pollution cannot be excluded as the amount of spherules in the sediments at the center was extremely high. The present study demonstrates that magnetometry has a practical application in detecting and mapping HM pollution in river systems.

## Introduction

Aquatic systems are one of the collectors of different pollutants, particularly heavy metals, which are likely to accumulate in water suspensions and the top layer of sediments. As a consequence of the increase in the population of cities and the urbanization process, the pollution of aquatic systems due to industrial wastewater discharges, sewage wastewater, fossil fuel combustion, and atmospheric deposition is becoming a large and critical issue on the global scale (Jordanova et al. 2004; Zhang et al. 2011).

The magnetic method (magnetometry) is one of the most effective tools for detecting and monitoring aquatic systems by measuring sediment properties. This method has been used in the study of the pollution levels of streams, rivers, lakes, and estuary sediments (Chan et al. 1997; Desenfant et al. 2004; Georgeaud et al. 1997; Horng et al. 2009; Hu et al. 2003; Li et al. 2011; Prajith et al. 2015; Scholger 1998; Wang et al. 2018).

The first parameter measured for assessing the pollution level is magnetic susceptibility (*χ*). Primarily, it is sensitive to the concentration of anthropogenic magnetic particles (AMP) that are accompanied by heavy metals. Magnetic susceptibility of river sediments is used for mapping areas exposed to industrial emissions from places such as coal-burning power plants, lead ore smelters, and sawmills (Jordanova et al. 2004; Petrovský et al. 2000; Prajith et al. 2015) and for tracing transport of pollutants (Frančišković-Bilinski et al. 2017). Many authors have reported that sediments rich in AMP are attributed to close sources of water pollution such as sawmills, paper industries, and sewage water cleaning farms (Petrovský et al. 2000); vicinity of iron ore loading docks and iron ore plants (Prajith et al. 2015); and the activity of industrial cities and harbors (Jordanova et al. 2004).

Magnetometry is also applied for tracing the transport of pollutants along the river by observing the migration of the strongly magnetic spherical-shaped particles (spherules) associated with heavy metals, which commonly originate from urban activities (power plants, smelting industries, disposal sites, municipal wastes) (Cowan et al. 2015; Desenfant et al. 2004; Li et al. 2011; Zhang et al. 2011). Changes in concentration-dependent magnetic parameters along core sediments can adequately reveal the distribution of river pollution over time. For instance, the maximum values of magnetic susceptibility were attributed to pollution episodes and periods of strong industrialization dating to the beginning of historic industrial activity (Chudaničová et al. 2016).

Moreover, magnetic methods can estimate the amount of AMP accompanied not only by heavy metals but also by some radionuclides, as magnetic susceptibility correlates with 232Th, 238 U, 40 K, and 226Ra radionuclides, presumably associated with the use of fertilizers in the surrounding agricultural areas (Chaparro et al. 2015; Krishnamoorthy et al. 2014; Suresh et al. 2011).

The present study aimed to evaluate the level of heavy metal pollution in Vistula river sediments within a highly urbanized area. The research was conducted in Warsaw agglomeration, which, in the central part of Poland, is the largest emitter of mainly urban pollution. The study was also designed to indicate what kind of activities of a big city impact the level of river pollution and how strongly urban pollution affects areas located outside the city.

Magnetic susceptibility was used to detect AMP accumulated in the top layer of sediments and for the evaluation of heavy metal pollution levels. Temperature changes of magnetic parameters and the hysteresis loops were used to identify the mineralogy of APMs and to discriminate the grain size of magnetic particles, respectively. Detailed magnetic analyses were complemented by scanning electron microscopy (SEM) observations to recognize the surface morphology of the magnetic particles and chemical element composition analysis to identify individual heavy metals.

## Study area

**Fig. 1:**
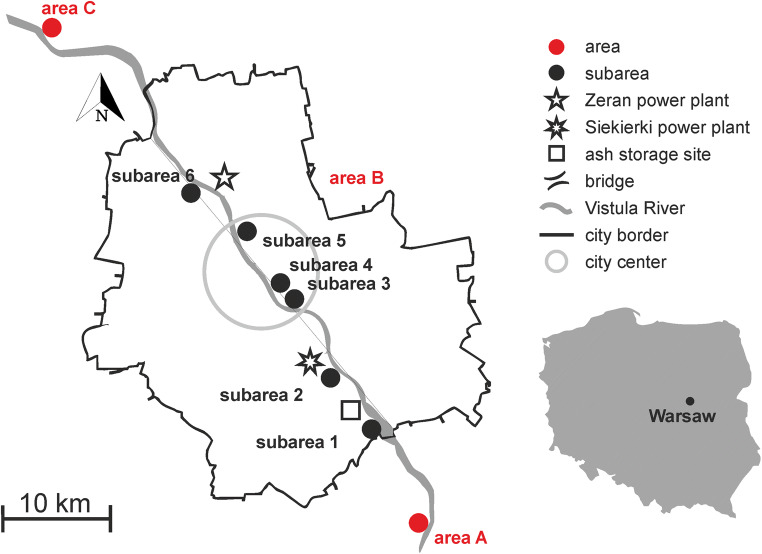
The map of Warsaw with sampling areas and subareas

The Vistula is the largest river in the Baltic Sea catchment and the longest in Poland. It flows from the mountains in southern Poland, through the two biggest cities Cracow in the southern part and Warsaw in the central part, and has the mouth to the Baltic Sea in the northern part of the country. The research was conducted in a 54-km section (approximately) of the Vistula River, including Warsaw agglomeration (area B) and two areas outside the city limits (areas A and C) (Fig. 1).

Area A is approximately 11 km up-river to the south of Warsaw in the vicinity of two cities, Konstancin-Jeziorna and Otwock, both smaller than Warsaw. This area is affected mainly by local pollution and is less polluted by pollutants typical of a large city. Area B located in Warsaw is mainly affected by urban activities due to the low levels of industrialization. The study was conducted in several subareas of area B (subareas 2–6, Fig. 1) located on the right or left bank of the river, depending on access to the riverbank and the possibility of sampling. Subarea 1 (part of area B, Fig. 1) was chosen as a reference area because it is away from any industrial and urban activities and represents the same sediment type as other areas. Riverbank sediments are lithologically uniform, comprising mainly Holocene sands and Pliocene silt and clay (Morawski 2011; Morawski and Pielach 2011; Sarnacka 1980a, b). Subarea 2 is near the largest power and heating plant in Warsaw (Siekierki station) and the power plant’s ash storage site. Subareas 3 and 4 are at the very center of the city on the unregulated left riverbank. Subareas 5 and 6 are approximately a few kilometers to the north of the city center, near the second largest power and heating plant (Żerań station) in Warsaw. The primary fuel in both power and heating plants is hard coal.

Area C is located down-river approximately 13 km to the north of Warsaw. There is no industrial or urban activity in area C or in its surroundings.

## Materials and methods

The collection of river sediments consisted of approximately 200 samples taken along the about 54-km section of the Vistula River in Warsaw in July–October 2016. A relatively low level and small flow velocity of water during sampling allowed for calm sedimentation of the currently polluting particles on the surface layer of sediments. Our preliminary study of the Vistula river sediments (Szczepaniak-Wnuk and Górka-Kostrubiec 2018) confirmed that surface sediments correspond to the maximum enhancement in magnetic particles and can reflect anthropogenic impact.

Top river sediments, called “surface samples,” were collected from each of areas A, B, and C. They were taken from approximately 0.5 m^2^ area up to ~2 cm thickness at the site where the water floods the riverbank. Samples were collected using a plastic shovel that was thoroughly cleaned in water and dried with a paper to avoid cross-contamination. The wet sediments (~0.5–1 kg) were put in plastic bags for transportation and storage. In laboratory, the samples were dried for few days at room temperature and then in an oven at approximately 50 °C for 3 h. Granulometric fractions with diameters between 1 and 0.5 mm (labeled as fraction 0.5), between 0.5 and 0.25 mm (labeled as fraction 0.25), between 0.25 and 0.1 mm (labeled as fraction 0.1), between 0.1 and 0.071 mm (labeled as fraction 0.071), and < 0.071 mm (labeled as fraction < 0.071) were obtained using a laboratory shaker M/LPzE-2e with a standard sieve set.

**Fig. 2:**
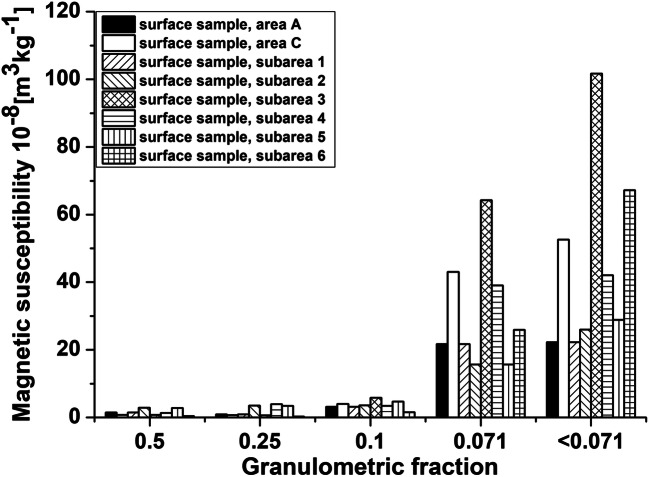
Distribution of magnetic susceptibility for individual granulometric fractions of surface sediments collected in subareas 4 and 5 (Szczepaniak-Wnuk and Górka-Kostrubiec 2018)

In our previous research, we studied the distribution of magnetic susceptibility in granulometric fractions for approximately 20 surface samples. In each area, the sediments showed the highest magnetic susceptibility in the finest fractions, i.e., 0.071 and < 0.071. Hence, the fine fractions 0.071 and < 0.071 were studied in this work. Figure 2 shows the distribution for exemplary samples taken from each area.

Magnetic susceptibility is a property that describes the capability of a substance to change its magnetization under the influence of an external magnetic field (Thompson and Oldfield 1986). This magnetic parameter provides information about the concentration and mineralogy of magnetic particles and the contribution of superparamagnetic particles (SPs). Low-field volume magnetic susceptibility (*κ*) was measured using the MFK1-FA multifunction kappa-bridge (AGICO, Czech Republic) at two magnetic field frequencies (976 and 1560 Hz) with an intensity of 200 A/m. Mass specific magnetic susceptibility (*χ*) (referred to as magnetic susceptibility for short and expressed as m^3^ kg^−1^) is normalized by a mass unit. The frequency dependence of magnetic susceptibility (*χ*_fd%_) was calculated as the percentage of magnetic susceptibility loss measured at low (976 Hz) and high (1560 Hz) frequencies of the magnetic field (Dearing et al. 1996).

The high-temperature changes of the magnetic susceptibility *κ*(*T*) curves are useful in rapid recognition of magnetic mineralogy by estimation of the Curie temperature, i.e., the temperature at which an ordered ferromagnetic phase transforms into a disordered paramagnetic phase. The *κ*(*T*) curves were measured in free air using a KLY-3 kappa-bridge (AGICO) with a CS-3 high-temperature furnace, at temperatures ranging from room temperature (*T*_R_) to 700 °C.

Low-temperature experiments were conducted for the < 0.071 fraction of the selected samples from area B by using an alternating current measurement system (ACMS, Quantum Design) and vibration sample magnetometer (VSM). The temperature changes of magnetization—curve of M(T)—were measured over the range 27 to − 262 °C during cooling in a magnetic field of 10 mT. The curve of thermal demagnetization of saturation remanence (SIRM(T)) was measured over the range of −262 to 27 °C in zero magnetic field. SIRM was acquired in the magnetic field of 2 T at temperature − 262 °C.

The shape and parameters of the hysteresis loop give information about magnetic mineralogy and domain structure of grains (grain size). The hysteresis loops were measured using an alternating gradient magnetometer with the vibration sample (VSM, MolSpin, UK). Saturation magnetization (*M*_s_), saturation remanent magnetization (*M*_rs_), and coercivity (*H*_c_) were determined from the hysteresis loops after subtracting the paramagnetic contribution. Both *M*_s_ and *M*_rs_ were mass-normalized. Remanence coercivity (*H*_cr_) was obtained from the curve of subsequent direct current (DC) back-field demagnetization of isothermal remanent magnetization (IRM).

Magnetic studies were supplemented by microscopy observations and chemical composition analysis. The shape and surface morphologies of magnetic particles were identified by SEM (JSM-6480, JEOL Akishima, Tokyo, Japan). The chemical composition of individual magnetic particles was determined using an energy dispersive X-ray spectrometer (EDS). The surface of the particles was analyzed by mapping the distribution and relative proportion (intensity) of chemical elements over the scanned area. The magnetic extract (magnetic particles) of sediments was separated using a neodymium hand magnet.

Concentrations of Pb, Zn, Cr, Co, Cu, Ni, Al, Fe, Mn, Ti, Cd, Ba, and As were determined using inductively coupled plasma mass spectrometry (ICP-MS, ELAN 6100 DRC, Perkin Elmer, USA). For chemical analysis, samples (0.15–0.20 g) were exposed to microwave digestion (Milestone UltraWAVE) in ~ 5 ml of 65% HNO_3_, with the temperature increasing by 12.5 °C min^−1^ to 270 °C, holding at 270 °C for 15 min, and then cooling down to room temperature. ICP-MS measurements and SEM observations with EDS analysis were conducted in standardized laboratories.

## Results

### Distribution of magnetic susceptibility of sediments

**Fig. 3:**
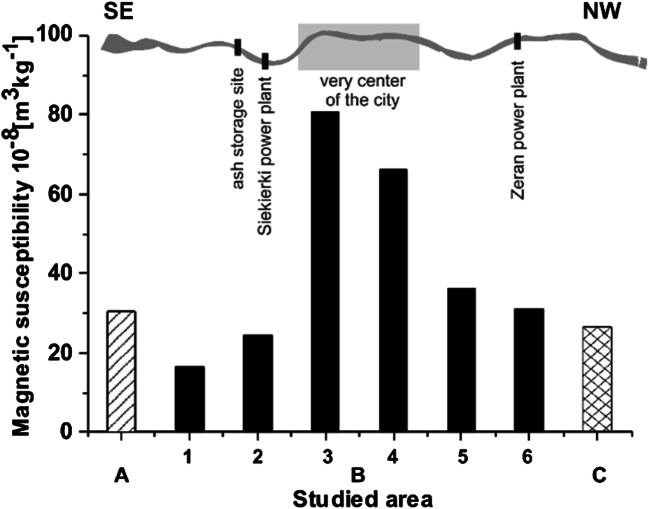
Distribution of average magnetic susceptibility (*χ*_av_) of the surface of Vistula river sediments for area A (column with striped pattern) located to the south of Warsaw, for subareas 1–6 (black columns) of area B located in Warsaw and for area C (column with grid pattern) located to the north of Warsaw

The distribution of average values of magnetic susceptibility (*χ*_av_) for fine grain fractions of surface sediments from A and C areas and from the six subareas of area B is shown in Fig. 3 and in Table 1. The *χ*_av_ is calculated as the sum of the volume magnetic susceptibility of all samples collected from individual areas divided by the sum of the sample masses. To the south of Warsaw (area A), the *χ*_av_ is ~ 30 × 10^−8^ m^3^ kg^−1^. In the subareas of area B, the values of *χ*_av_ vary as follows: ~ 17 × 10^−8^ m^3^ kg^−1^ in subarea 1 and ~ 24 × 10^−8^ m^3^ kg^−1^ in subarea 2. In the very center of city, *χ*_av_ reaches a maximum of ~ 80 × 10^−8^ m^3^ kg^−1^ in subarea 3 and ~ 66 × 10^−8^ m^3^ kg^−1^ in subarea 4, and then decreases to ~ 36 × 10^−8^ m^3^ kg^−1^ for subarea 5 and to ~ 30 × 10^−8^ m^3^ kg^−1^ for subarea 6. In area C located to the north of Warsaw, *χ*_av_ is relatively low with the value of ~ 26 × 10^−8^ m^3^ kg^−1^.

**Table 1:** Average magnetic susceptibility (*χ*_av_) of the surface of Vistula river sediments for area A, for subareas 1–6 of area B and for area C with statistical parameters

*Χ*_av_ 10^−8^m^3^kg^−1^						
	Number of samples	Minimum values	Maximum values	Average values	Median	Standard deviation
Area A	5	24.0	38.0	30	30	4
Subarea 1	6	10.1	28.4	17	17	4
Subarea 2	19	8.3	52.3	24	24	11
Subarea 3	9	25.9	189.7	80	70	38
Subarea 4	14	20.4	106.2	66	55	22
Subarea 5	22	10.0	110.4	36	30	25
Subarea 6	13	13.2	89.0	30	31	19
Area C	8	14.7	52.6	26	27	10

**Fig. 4:**
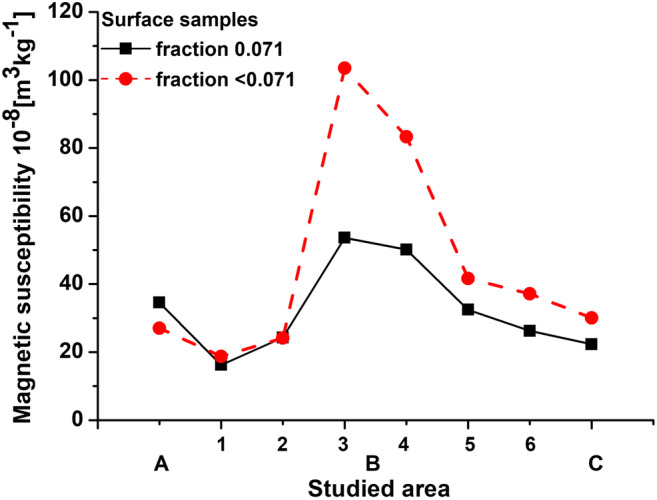
Distribution of average magnetic susceptibility for the fine (*χ*_av″0.071″_) and finest (*χ*_av″ < 0.071″_) granulometric fractions of surface sediments of the Vistula river for area A, subareas 1–6 of area B and for area C

In the very center of the city, the finest fraction reveals almost two times higher values of average magnetic susceptibility (*χ*_av″ < 0.071″_) than fraction 0.071 (see Fig. 4), which indicates the emission of fine-grain magnetic particles from sources typical of urban activities. For areas located to the north of the city center, *χ*_av″ < 0.071″_ exceeds *χ*_av”0.071″_, which could be the result of transfer of the finest magnetic particles from the city center. This is confirmed by the results obtained for reference subarea 1 and subarea 2 located in the south of Warsaw, for which values of *χ*_av_ do not differ significantly for both fractions. Moreover, the higher value of *χ*_av_ for the 0.071 fraction relative to the < 0.071 fraction obtained in area A located approximately 20 km to the south of Warsaw can be related to the influence of slightly different sources of AMP than those in the center of Warsaw (subareas 3 and 4). Area A is located in the vicinity of two cities, which probably, due to their smaller area than Warsaw, generate lesser amount of particles typical for big urban agglomerations. This is apparent by higher *χ*_av_ values for the 0.071 fraction than those for the fraction of < 0.071.

### Thermomagnetic analyses

**Fig. 5:**
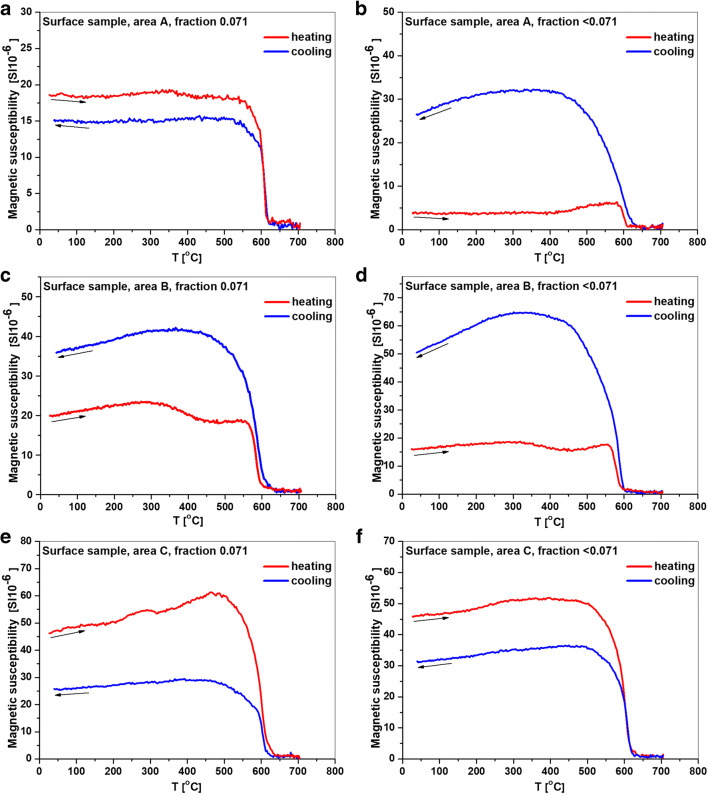
Changes in magnetic susceptibility curves during heating and cooling-*κ*(*T*) for the 0.071 and < 0.071 fractions of sediments from areas A (**a**, **b**), B (**c**, **d**), and C (**e**, **f**)

For area B (Fig. 5c and d), all the heating curves indicate magnetite, with a Curie temperature of ~ 580 °C, as the main ferromagnetic phase for both 0.071 and < 0.071 fractions. The slight decrease in magnetic susceptibility observed between ~ 300 and 450 °C is usually interpreted as the transformation of magnetically strong and metastable maghemite to hematite that has lower κ (Dunlop and Özdemir 1997). In both fractions, the cooling curves below 580 °C run above the heating curves, indicating thermally induced transition of weakly magnetic minerals, mainly paramagnetic, into strongly magnetic minerals such as magnetite. However, for the 0.071 fraction, the cooling curves (Fig. 5c) start to increase at a slightly higher temperature than during heating. This could be the effect of formation of the new cation-deficient magnetite in the samples during heating.

**Fig. 6:**
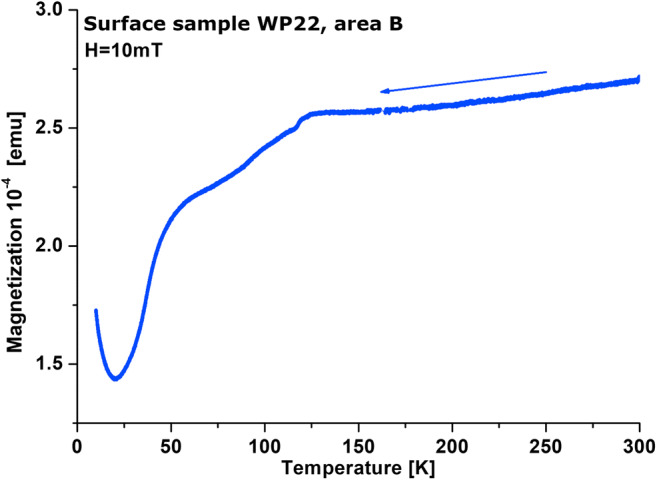
Exemplary low-temperature cooling curve of *M*(*T*) for a sample from area B

The low-temperature technique offers a complementary tool for the identification of stoichiometric magnetite by observing structural transition, the so-called Verwey transition. It is a first-order crystallographic transition from the cubic to the monoclinic phase at T_v_ between − 163 and − 153 °C. As the low-temperature measurement, in contrast to the high-temperature experiment (above *T*_R_), does not cause permanent chemical alteration (Moskowitz 1991), the M(T) curve was measured at low temperatures in the present study. The cooling curve of M(T) for area B (Fig. 6) showed the Verwey transition at ~ − 153 °C, confirming the presence of magnetite.

For areas A and C, all the heating curves of κ(T) show the Curie temperatures between 600 and 615 °C. This can be indicative of the presence cation-deficient magnetite as the primary magnetic phase in sediments from these areas. As reported by Dunlop and Özdemir (1997) and Tauxe (2005), the partial oxidation of magnetite leads to loss in the volume of the lattice structure of magnetite, resulting in characteristic cracking of the surface, loss in magnetization, and increase in the Curie temperature in comparison with stoichiometric magnetite.

Only for a few samples from areas A and C, the course of the cooling curve is the same as that for sediments from area B, i.e., they are above the heating curves. In most cases, the value of *κ* after the heating-cooling cycle is lower than the initial value before heating. According to Jordanova et al. (2012), this phenomenon can be explained by the oxidation of cation-deficient magnetite to weakly magnetic minerals during heating.

### Hysteresis parameters

**Fig. 7:**
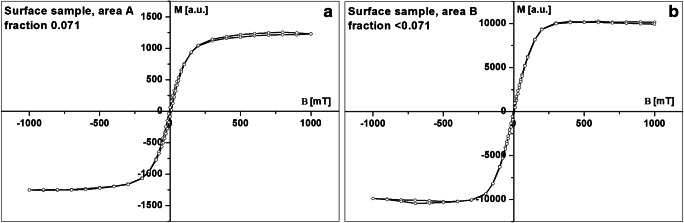
Examples of hysteresis loops after subtracting paramagnetic contribution for sediments taken from area A: fraction 0.071 (**a**) and from area B: fraction < 0.071 (**b**)

In all investigated areas, the hysteresis loops are relatively narrow and saturated at relatively low magnetic fields of approximately 300–350 mT. The number of measured samples and the statistical parameters are presented in Table 2. The narrow hysteresis loops (Fig. 7) and values of *B*_c_ (Table 2) confirmed the results of the thermomagnetic study, as soft magnetic minerals such as magnetite and/or maghemite are the main magnetic phase.

**Table 2:** Average concentrations of heavy metals and PLI index for area A, subareas 1–6 of area B, and area C

***M***_**s**_**10**^**−3**^**Am**^**2**^ **kg**^**−1**^**fraction 0.071**				***M***_**s**_**10**^**−3**^**Am**^**2**^ **kg**^**−1**^**fraction < 0.071**		
	**Area A**	**Area B**	**Area C**	**Area A**	**Area B**	**Area C**
Number of samples	4	35	4	4	34	4
Minimum values	52.3	14.5	80.5	87.4	17.7	130.2
Maximum values	222.3	356.3	295.8	254.6	136.6	566.8
Average values	158.8	78.2	171.1	157.5	54.9	276.0
***M***_**rs**_**10**^**−3**^**Am**^**2**^ **kg**^**−1**^**fraction 0.071**				***M***_**rs**_**10**^**−3**^**Am**^**2**^ **kg**^**−1**^**fraction < 0.071**		
Number of samples	4	35	4	4	34	4
Minimum values	4.4	0.9	5.0	4.3	1.2	5.2
Maximum values	12.4	32.6	25.0	12.2	8.8	41.4
Average values	9.6	5.9	14.0	8.10	4.1	20.5
***B***_**c**_**[mT] fraction 0.071**				***B***_**c**_**[mT] fraction < 0.071**		
Number of samples	4	35	4	4	34	4
Minimum values	3.0	2.0	4.0	5.5	3.0	5.5
Maximum values	6.5	10.5	9.0	8.5	10.5	7.0
Average values	5.3	5.3	6.1	7.0	7.1	6.4
***B***_**cr**_**[mT] fraction 0.071**				**B**_**cr**_**[mT] fraction < 0.071**		
Number of samples	4	35	4	4	34	4
Minimum values	12.0	4.0	20.0	22.0	7.0	16.0
Maximum values	46.0	43.0	27.0	40.0	45.0	27.0
Average values	27.7	26.0	23.0	33.3	28.5	24.0

**Fig. 8:**
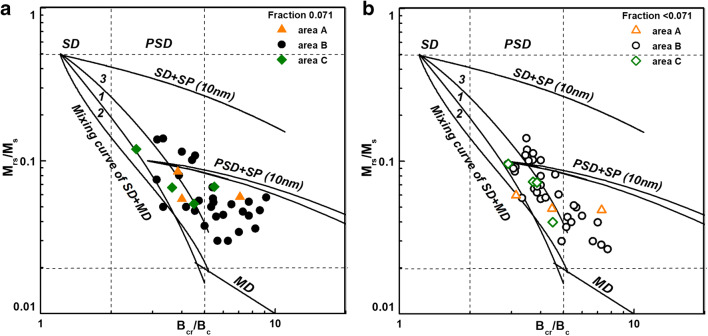
Ratios of hysteresis parameters for 0.071 (**a**) and < 0.071 (**b**) fractions of sediments on Day-Dunlop plot

**Fig. 9:**
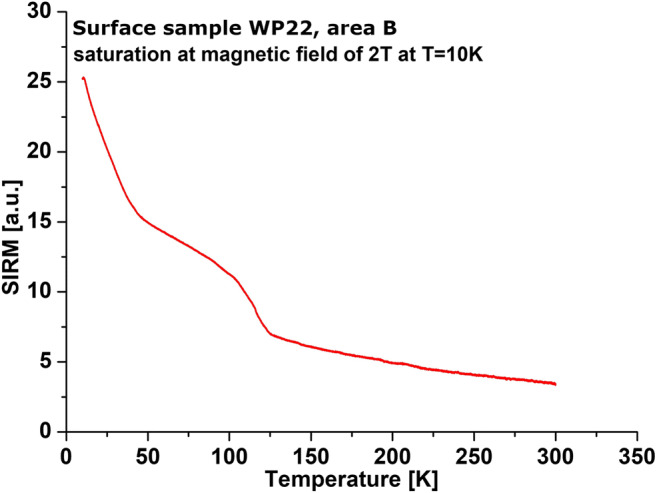
Curve of thermal demagnetization of SIRM acquired at − 267 °C in a magnetic field of 2 T (**b**) for selected sediment samples from area B

Figure 8 shows the ratios of hysteresis parameters on the Day-Dunlop plot (Day et al. 1977; Dunlop 2002). The Day plot demarcates areas with values of hysteresis ratios of *M*_rs_/*M*_s_ and *B*_cr_/*B*_c_ as follows: *M*_rs_/*M*_s_ > 0.5 and *B*_cr_/*B*_c_ < 2 for single-domain (SD) magnetite (grain size 0.03–0.08 μm), *M*_rs_/*M*_s_ < 0.02 and *B*_cr_/*B*_c_ > 5 for multi-domains (MD) (grain size > 20 μm), and *M*_rs_/*M*_s_ = 0.02–0.5 and *B*_cr_/*B*_c_ = 2–5 for pseudo-single domains (PSD) (grain size 0.1–20 μm). Additionally, Dunlop (2002) empirically constructed three curves for mixtures of SD + MD grains, mixtures of SD + SP (10 nm) grains, curve for MD grains, and curve for mixtures of PSD + SP (10 nm) grains. Regardless of the sampling areas, most of the samples of the 0.071 fraction lay between the “3” mixing curve of SD + MD elongated magnetite grains and the mixing curve for PSD + SP (10 nm grains) (Fig. 8a). For the < 0.071 fraction (Fig. 8b), almost all samples are located along the “3” mixing curve of SD + MD elongated magnetite grains. The frequency-dependent magnetic susceptibility parameter is used for estimating the contribution of SP. However, different grain size ranges are sensitive to different frequencies of the magnetic field. The MFK1-FA Kappa-bridge (AGICO) operates in the magnetic field frequencies sensitive to SP at the grain size of 15–25 nm. According to Dearing plot (Dearing et al. 1996), the values of *χ*_fd%_, from ~ 1 to ~ 7% for the 0.071 fraction and from ~ 1 to ~ 6% for the < 0.071 fraction indicate a little more than 10% contribution of SP grains of size 15–25 nm. To obtain information about the presence of SP grains with wider distribution of grain size, the SIRM(T) curves were measured at low temperatures (Fig. 9). During heating from − 267 to − 233 °C, the SIRM curve shows rapid hyperbolic decrease typical of superparamagnetic behavior, which can be ascribed to the distribution of blocking temperatures due to the wide grain size of SP particles (King and Williams 2000). A very well visible inflection point at the Verwey transition of ~ − 153 °C clearly confirms the presence of almost ideal stoichiometry magnetite (O’Reilly 1984).

### Morphology

**Fig. 10:**
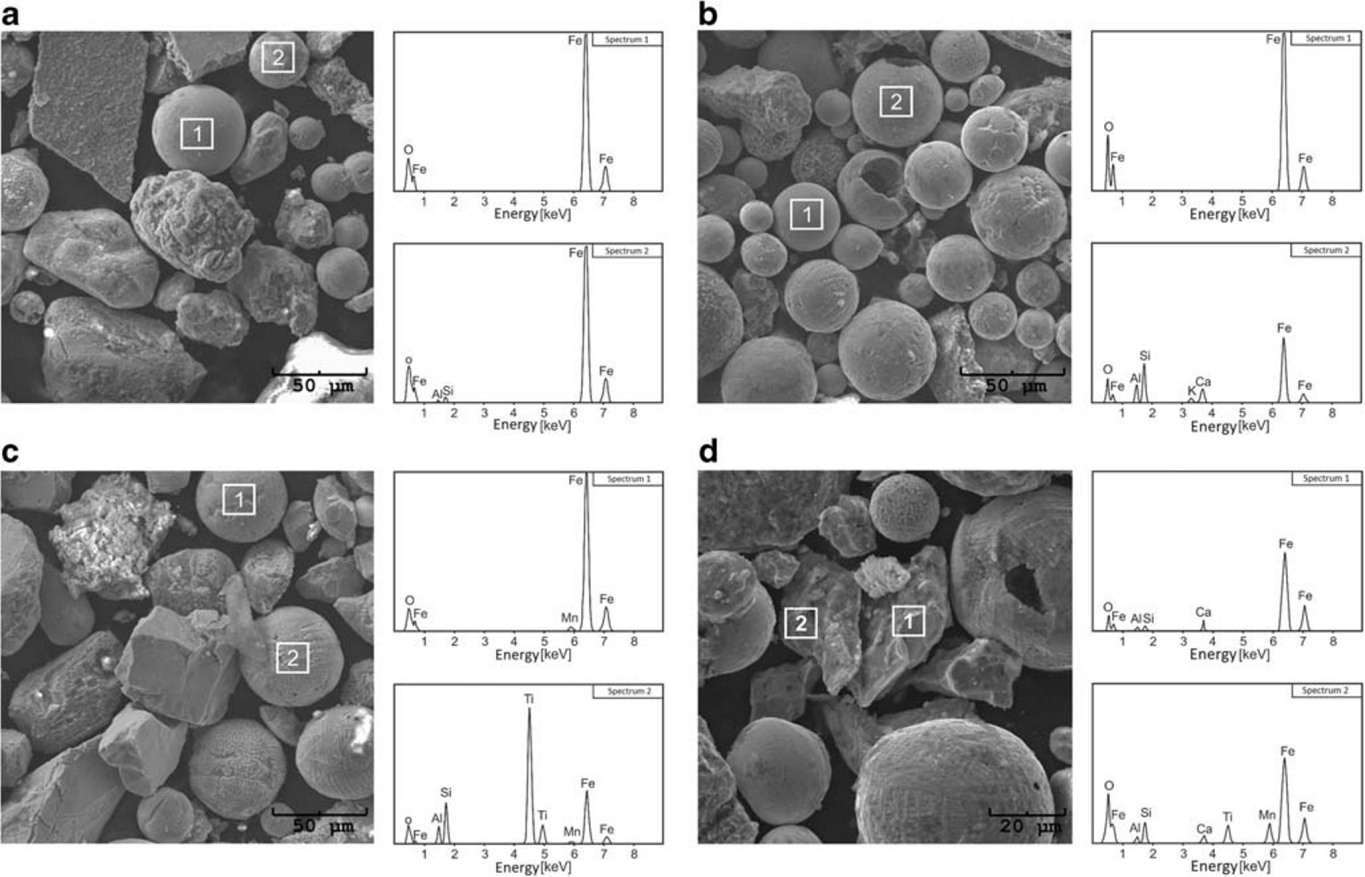
SEM images and chemical composition (EDS spectra) for magnetic extracts of <0.071 fraction: area A (a), area B (b), and area C (c, d)

The shape and surface morphologies of magnetic particles are identified for magnetic extracts of 9 samples from area B, 3 samples from area A, and 3 samples from area C. SEM observations reveal spherical and irregular particles in magnetic extract in both fine fractions (Fig. 10a–d). The largest amount of spherules (~ 80–90% of the magnetic extract) with a diameter varying from a few micrometers to ~ 100 μm is observed at the city center. In areas outside the city center (subareas 2, 5, and 6) and out of city limits (areas A and C), the amount of spherules is significantly lower or comparable with the amount of irregular particles. Individual spherules differ in surface morphology, such as “orange peel,” hexagonal pattern, and thread-like features, similar to those recognized by Jordanova et al. (2004). EDS analysis shows that the spherules mainly consist of iron oxides, Al, Si, and additional amounts of Mn, Ca, and K. In area B, irregular particles constitute ~ 10–20% of the magnetic extract, whereas in areas A and C, the amount of these particles is comparable or higher. Irregular particles also differ in shape (with angular or round ridges). Their size ranges from a few to ~ 200 μm, and they consist of iron oxides, Al, Si, Ca, Zn, Mn, and Ti.

### Heavy metal concentration

**Fig. 11:**
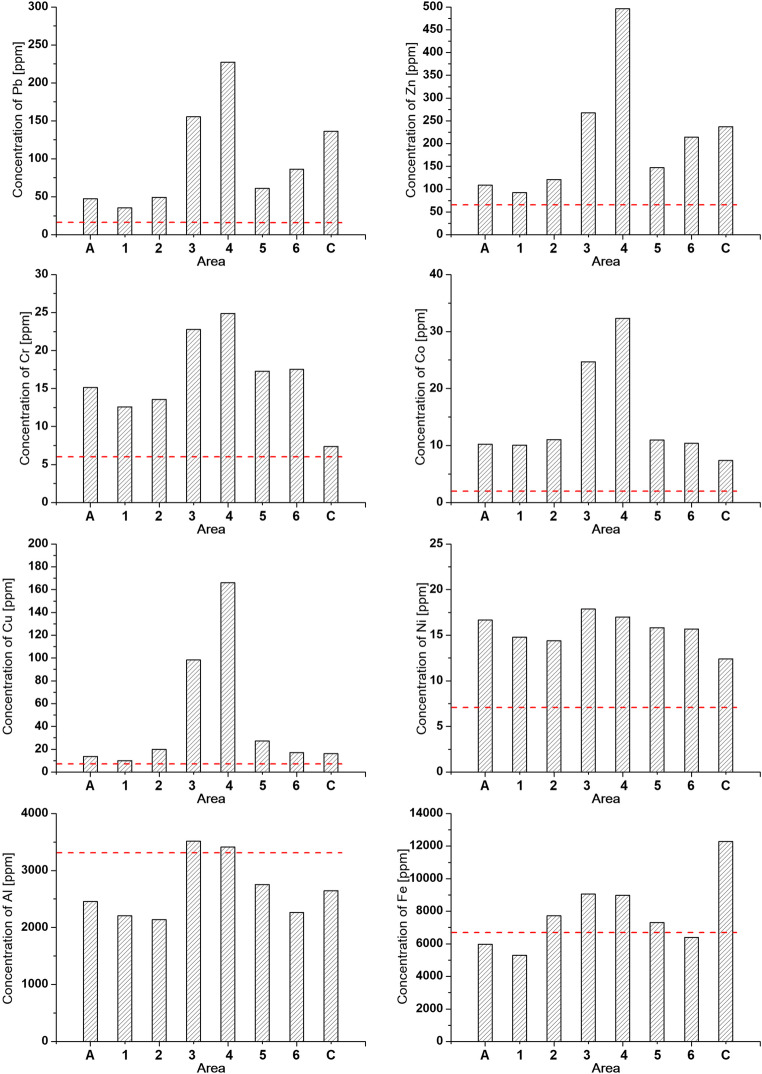

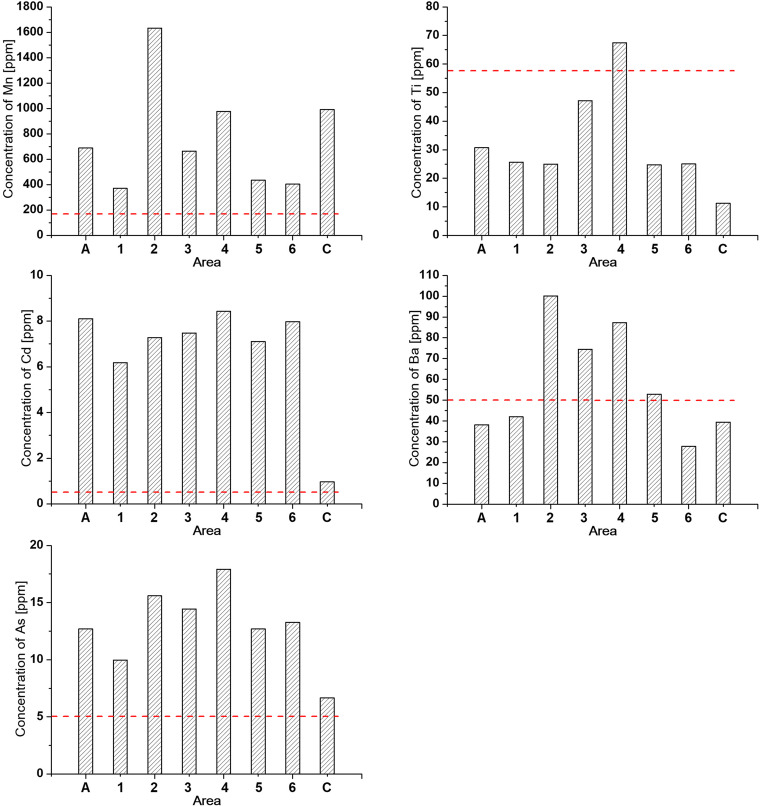
Concentrations of selected heavy metals for <0.071 fraction of sediments for area A, subareas (1–6) in area B, and area C

As the fraction < 0.071 has the highest *χ* and, by inference, is rich in AMP, we determined the heavy metal concentrations only for this fraction. The distributions of Pb, Zn, Cr, Co, Cu, Ni, Al, Fe, Mn, Ti, Cd, Ba, and As concentrations are shown in Fig. 11 and listed in Table 3. The red dashed line in Fig. 11 represents the background values of heavy metal concentrations for Polish sediments from Geochemical Atlas of Warsaw and the surrounding area (Tomassi-Morawiec 2016).

**Table 3:** Hysteresis loop parameters for 0.071 and < 0.071 fractions of sediments

Element (ppm)	Granulometric fraction of < 0.071							
	A	1	2	3	4	5	6	**C**
Pb	47.1	35.6	49.3	155.3	227.4	60.9	86.1	136.3
Zn	109.0	92.5	121.5	267.4	496.3	147.2	214.4	237.3
Cr	15.1	12.6	13.6	2.8	24.9	17.3	17.5	7.4
Co	10.2	10.1	11.1	24.7	32.3	10.9	10.4	7.4
Cu	13.8	10.1	19.8	98.3	165.9	27.2	17.1	16.1
Ni	16.6	14.8	14.4	17.9	17.0	15.8	15.7	12.4
Al	2455.1	2205.7	2138.8	3516.1	3410.7	2754.8	2262.4	2646.8
Fe	5966.1	5291.7	7714.5	9058.7	8970.5	7305.6	6395.1	12,280.9
Mn	689.0	371.7	1633.0	664.8	976.6	435.2	403.8	992.0
Ti	30.7	25.7	25.0	47.1	67.5	24.7	25.0	11.3
Cd	8.1	6.2	7.3	7.5	8.4	7.1	7.9	0.9
Ba	38.2	42.0	100.1	74.5	87.4	52.9	27.8	39.5
As	12.7	9.9	15.6	14.4	17.9	12.7	13.3	6.7
PLI	2.3	1.9	2.7	4.0	3.0	2.5	2.3	1.9

In area A, the concentrations of Pb, Zn, Cr, Co, Cu, Ni, Mn, Cd, and As are higher than those for the background. The largest increase is observed for Co and Cd concentrations, i.e., ~ 5 times and ~ 8 times, respectively. The concentrations of Al, Fe, Ti, and Ba are slightly lower than those of the background. In the very center of Warsaw, the concentrations of heavy metals increase, reaching a maximum and then decrease as the distance from the center increases. The largest increase in subareas 3 and 4 is observed for Cu (~ 24 times), Pb (~ 15 times), Co (~ 17 times), Zn (~ 10 times), Cd (~ 8 times), Mn and Cr (~ 5 times), and As (~ 4 times). The smallest difference in relation to background is observed for concentrations of Ni, Al, Fe, Ti, and Ba, which are only ~ 1.5 to ~ 2 times higher.

One of the approaches to analyze the contribution of heavy metals to the overall toxicity of sediments is to use the Tomlinson Pollution Load Index (PLI) (Tomlinson et al. 1980). PLI is defined as the geometric mean of the concentration factor (CF_*n*_):

$\mathrm{PLI}=\sqrt[n]{{\mathrm{CF}}_1\cdot {\mathrm{CF}}_2\cdot \dots \cdot {\mathrm{CF}}_n}\sqrt[\mathrm{n}]{{\mathrm{CF}}_1\cdot {\mathrm{CF}}_2\cdot \dots \cdot {\mathrm{CF}}_{\mathrm{n}}}$,where ${\mathrm{C}\mathrm{F}}_n=\frac{{\mathrm{C}}_n}{{\mathrm{C}}_n(bg)}$ is the ratio of the concentration of the *n*th heavy metal (C_*n*_) to its corresponding background value (C_*n*(bg)_). The PLI shows how much the concentrations of heavy metals for particular samples exceed the background value; it is approximately 1 if the elemental load is near the background level and > 1 if the environment is exposed to metal toxicities (Tomlinson et al. 1980). According to Singh et al. (2003), the following PLI ranges are used to estimate the level of pollution: 1 < PLI < 2 for moderately polluted, 2 < PLI < 3 for highly polluted, and PLI > 3 for extremely polluted. To calculate PLI, the geochemical background values of heavy metals for Polish sediments are used (Geochemical Atlas of Warsaw and the surrounding area; Tomassi-Morawiec 2016). We think that these background values best reflect the natural level of heavy metals in unpolluted river sediments. In areas located outside the city limits (A and C) and subareas outside the city center (subareas 2, 5, and 6), the sediments are highly polluted as the values of PLI range between 2 and 3. Higher values of PLI, ~ 3 to ~ 4, at the very center of the city, point to extremely strong pollution of sediments in this area (Table 3). This result agrees with the distribution of magnetic susceptibility as well as the concentrations of individual heavy metals.

## Discussion

In the subareas of area B, the changes in the distribution of magnetic susceptibility and concentration of heavy metals in sediments indicate a high level of pollution and a strong impact of anthropogenic factors associated with the activities of city. The impact of local sources on the level of sediment pollution is apparent in the distribution of *χ*_av_ along the river, as the values of *χ*_av_ at the city center are approximately four times higher than those obtained for the reference subarea 1. A similar distribution trend is noted for heavy metals, i.e., Cu, Pb, Co, Zn, Cd, Mn, Cr, and As. In relation to the background, their concentrations increase over a dozen times, with decreasing distance from the southern subareas to the center.

The very center of the city seems to be the most polluted part of the river, and it relatively strongly affects areas located in the north. A high concentration of magnetic particles and almost 80% contribution of spherical particles to magnetic extracts are characteristics for the finest (< 0.071) fraction. This result is confirmed by studies of the Arc River in France (Desenfant et al. 2004), which showed that the fraction with a grain size below 63 μm is enriched in spherical AMP. The thermomagnetic curves supplemented by relatively narrow hysteresis loops, which saturated in a field of ~ 300–350 mT, indicate magnetite as the dominant magnetic phase. It is well known that magnetite is typical mineralogy of particles formed by the anthropogenic process in urban areas (Brown et al. 2011; Flanders 1994; Jordanova et al. 2004; Kukier et al. 2003). The distribution of hysteresis parameters on Day-Dunlop plot indicates the mixture of SD grains with approximately 70% contribution of MD grains in the finest fraction. As reported by Górka-Kostrubiec et al. (2014) and Górka-Kostrubiec et al. (2012), it is characteristic for pollution accumulated in urban dust, soil, and air filters in Warsaw agglomeration. For fine fraction of 0.071 the mixture of PSD grains with small contribution of SP grains is identified from the Day-Dunlop plot and the low temperature curve of SIRM(T). The SP with a wide range of grain size could originate from weathering and erosion processes of naturally formed lithogenic riverbed (Nguyen et al. 2016).

The most frequently observed particles in the finer-grained magnetic extract of the Vistula sediments are spherules with different surface morphologies and diameters ranging from a few to ~ 100 μm. Many studies show that in urban areas, the magnetic spherules in the size range 0.001–90 μm are usually produced in coal burning processes (Jordanova et al. 2006; Kukier et al. 2003; Kutchko and Kim 2006; Magiera et al. 2011). The spherical particles are mainly derived from fossil fuel combustions in heat and power plants, and they are very often found in fly ash, top soils, and indoor and outdoor air. Magiera et al. (2011) reported that polluted industrial soils contain spherical particles enriched in magnetite/maghemite that originated from burning coal. Spherical particles were also observed by Petrovský et al. (2013) in PM10 daily samples collected at sites with different degrees of atmospheric pollution and by Górka-Kostrubiec (2015) in the finest fraction of indoor dust in Warsaw agglomeration. Surface morphology and chemical composition of individual spherules from sediments collected in the vicinity of major cities along the Danube River were studied by Jordanova et al. (2004). The authors showed that industry-origin spherules have very diverse surface morphologies such as orange peel, dendritic structure, hexagonal pattern, and thread-like structure; strong magnetic properties; and are often associated with various chemical elements, a.o. iron oxides, Si, Ni, and Cu.

In Vistula river sediments, SEM observations reveal several types of surface morphology of spherules: smooth, wrinkled, and with mineralization on the surface. In addition to the typical peaks for iron oxides, Al, and Si, the peaks for Mn, Ca, and K are also identified on EDS spectra. The presence of magnetite-like spherical particles in < 0.071 fraction may indicate the strong influence of local sources on the pollution level in the center of Warsaw. Because Warsaw is not a highly industrialized area, the magnetite-like spherules produced in high-temperature processes may be the effect of emissions from two heating and power plants operating in the limits of the city. A significant share in the emission of magnetic spherules in Warsaw is that of low-stack emission, i.e., fireplaces and boiler rooms in household and public buildings in which solid and liquid fuels (gas, coal, and coke) are burned. These emissions can also contribute to the large amount of spherules observed in the surface of sediments and river water in the city center. As fine spherules transferred indoor by air were observed in indoor dust collected from apartments in the center of Warsaw (Górka-Kostrubiec 2015), it is very likely that spherules in sediments and water originate from atmospheric air.

The angular particles with sharp or round ridges are formed in processes related to the movement of vehicles and the abrasion of road surfaces (Bućko et al. 2010, 2011, 2013; Kim et al. 2007; Maher et al. 2008; Moreno et al. 2003; Muxworthy et al. 2001). Thus, it may be concluded that moving vehicles could be the source of angular particles often associated with Pb, Zn, and Cu, which are observed in the Vistula sediments. In Warsaw area, traffic-related pollution can enter the river in the form of dry and/or wet precipitation from the main routes on both banks and from high-traffic road bridges. Traffic-related pollution enriched in Cu, Pb, and Zn metals together with storm wastewater can also enter the river as sludge from stormwater drains. Unlike municipal and industrial wastewater, the storm wastewater is not treated in water purification plants to neutralize heavy metals and other hazardous substances to protect the aquatic ecosystem. Adamiec (2017) studied the heavy metal content in road dust, sludge from storm drains, and roadside soil from Warsaw by using chemical analysis combined with chemical fractionation. The author showed that in < 20-μm grain fraction of the street dust, the concentration of Zn and Cu exceeded 15–18 times and 6–14 times the background value on average, respectively. Among road dust, sludge, and roadside soil, the highest concentration of Pb was identified in the sludge. Zn and Cu mainly originate from brake and tire wear, while Pb is an important component of bearing alloys and is commonly used as the material for wheel balancing weights in cars (Adamiec 2017; Haus et al. 2007; Hjortenkrans et al. 2007; Schauer et al. 2006).

Other sources of angular particles could also be wearing of constructions, gutter outlets, and sawmills that contribute to increase in magnetic susceptibility as well as the pollution levels of river sediments (Petrovský et al. 2000). The highest concentrations of Mn, Cd, Ba, and As observed in subarea 2 in Warsaw may be related to contemporary and historical sources of pollution transported by the Wilanówka River flowing into the Vistula River in this subarea. The presence of Mn, Cd, Ba, and As in the waters and sediments of Wilanówka is linked with a paper mill operated earlier in this area (Geochemical Atlas of Warsaw and the surrounding area 2016). The Wilanówka stream reservoir is supplied by the waters of Służew Stream and Wilanów Lake into which wastewater is discharged, especially from the Służew industrial area in the past and from the airport at present. Currently, the water sediments of the Służew Stream are still enriched with heavy metals such as Cr, Cu, Pb, Ni, Cd, and Hg as their concentrations exceed the values of the geochemical background (Bojakowska et al. 2014). The sediments of Wilanówka may act as a potential source of secondary pollution of the water and sediments of the Vistula river, e.g., by remobilization of heavy metals due to the active fluvial processes and changes in geochemical conditions.

In areas A and C, there are differences in the distribution of magnetic particles in the fine granulometric fractions of sediments. In area A, the highest concentration of magnetic particles is in the fraction of 0.071, whereas in area C, most of the magnetic particles are present in the fraction of < 0.071. However, the magnetic fraction of sediments from both areas reveals similar mineralogy and domain state of magnetic grains. The fraction < 0.071 contains the mixture of SD grains with approximately 70% contribution of MD, whose mineralogy is dominated by cation-deficient magnetite, whereas the fraction of 0.071 contains PSD grains with SP particles in relatively wide range of grain size. In contrast to the highly polluted part of the city center, the sediments from areas A and C consist of irregular-shaped particles with angular or round ridges.

The local impact of pollution sources for area A is caused by the activity of two cities, namely Konstancin-Jeziorna and Otwock, located ~ 20 km to the south of Warsaw. Both cities are significantly smaller than Warsaw, and they are likely to generate less pollution, which enter the river. Lesser anthropogenic impact of both cities results in lower values of magnetic susceptibility, lower contribution of magnetic particles to the finest granulometric fraction, and lower content of spherules than those in Warsaw area (Fig. 4), and it does not significantly affect the river sediments located below the cities (subarea 1). Although Konstancin-Jeziorna and Otwock are less urbanized and populated than Warsaw, their activities generate relatively large amounts of heavy metals such as Cd, As, Mn, Ni, and Cr that can be considered as a potential hazard for the aquatic ecosystem.

With the increase in distance from the city center to the north (toward area C), the values of *χ*_av_ decrease to approximately two times within a short ~ 11-km section of the river. These changes in magnetic susceptibility are accompanied by a decrease in the concentration of individual elements such as Pb, Zn, Cr, Al, Fe, Cd, Ba, and As and the values of PLI. However, the values of *χ*_av_ and the concentrations of heavy metals remain higher than those in the south of Warsaw (area A), suggesting the transfer of magnetic particles together with heavy metals from the heavily polluted central part of the city. As area C is slightly urbanized and industrialized, the only source of pollution may be particles carried by the river from the city center. The rapid decrease in *χ*_av_ and heavy metal concentrations within a short and unregulated ~ 11 km section of the river could be explained by self-purification of the Vistula water. The ability of self-purification of the river depends on the effectiveness of the biochemical, hydrochemical, and physical processes. Biochemical processes lead to the degradation and decomposition of organic wastes by microorganisms and plants in the water and riverbank. The biochemical processes are controlled by the access to oxygen and diversity of ecological microhabitats. Such conditions are characteristic of unregulated river. A self-cleaning capacity of unregulated river can be many times greater than that of a regulated river. The intensity of hydrochemical processes such as oxidation and mineralization depends on the presence of clays, silts, colloidal particles, and Mn and Fe oxides and hydroxides in water and sediments (Ugwu and Igbokwe 2019; Vagnetti et al. 2003) and coexist with physical processes such as adsorption, dilution, and sedimentation. For area C, all these processes could be responsible for the down-river decrease of the concentration of magnetic particles, resulting in the decrease of the magnetic susceptibility in sediments.

## Conclusions

Our research is focused on the assessment of heavy metal pollution levels of the Vistula River sediments and the indication of urban activities (urban sources) affecting this pollution level. The strongest anthropogenic impact is observed in the center of the city, where the magnetic susceptibility, concentration of individual heavy metals, and PLI reach the maximum values in the finest faction < 0.071 mm. The most polluted sediments in the area of city are dominated by magnetite-like spherules with diverse surface morphologies, i.e., smooth, wrinkled, and with mineralization on the surface.

In the city center, we found that the concentrations of Zn, Cu, and Pb in sediments could be linked to the traffic-related sources generating angular-shaped magnetic particles. The spherical-shaped magnetite-like particles originate mainly from the operation of the heat and power plants and low-stack emission from the individual heating systems. It is very likely that the majority of traffic-related angular particles are discharged into the waters of the river reservoir by sludge from stormwater drains, while spherules are transported mainly as dry precipitate by air. However, we believe that this is not the only source of such a large number of spherules in the city center sediments. It is very likely that another source supplies spherical particles directly to the river; however, this assumption needs to be confirmed by more detailed studies and requires the collection of new samples.

In the area out of the city limits (area A), the decrease in magnetic susceptibility and heavy metal concentrations in the finest granulometric fraction in relation to the city center indicates lower pollution level. The activities of poorly urbanized and small populated cities are responsible for low pollution level assessed in area A. However, even such limited urban activity can generate relatively high amount of heavy metals such as Cd, As, Mn, Ni, and Cr, which are harmful for the aquatic ecosystems. In this area, the angular-shaped magnetic particles consist of cation-deficient magnetite that primarily contribute to the 0.071 mm granulometric fraction of sediments.

Area C located down-river is highly affected by pollutants transported from the city center; this is because for the finest fraction < 0.071 mm, the increasing distance from the source of pollution is accompanied by a systematic decrease in magnetic susceptibility and concentration of heavy metals. The conditions of unregulated riverbank and vegetation richness (biodiversity) between city center and area C intensify the physical and biochemical processes, resulting in relatively effective self-purification of the river.

The study shows that the changes in magnetic susceptibility and heavy metal concentrations and the correlations between them enable to use the magnetic methods as a tool for the assessment of heavy metal pollution of sediments in highly urbanized areas.

## References

[CR1] Adamiec E (2017). Chemical fractionation and mobility of traffic-related elements in road environment. Environ Geochem Health.

[CR2] Bojakowska I, Lech D, Jaroszyńska J (2014). Heavy metals in sediments of water bodies in the Służew stream catchment (Warsaw area). Environ Protect Nat Resour.

[CR3] Brown P, Jones T, Bérubé K (2011). The internal microstructure and fibrous mineralogy of fly ash from coal-burning power stations. Environ Pollut.

[CR4] Bućko MS, Magiera T, Pesonen LJ, Janus B (2010). Magnetic, geochemical, and microstructural characteristics of road dust on roadsides with different traffic volumes- case study from Finland. Water Air and Soil Pollution.

[CR5] Bućko MS, Magiera T, Johanson B, Petrovský E, Pesonen LJ (2011). Identification of magnetic particulates in road dust accumulated on roadside snow using magnetic, geochemical and micro-morphological analyses. Environ Pollut.

[CR6] Bućko MS, Mattila OP, Chrobak A, Johanson B, Cuda J, Tucek J, Zboril R, Pesonen LJ, Leppäranta M (2013). Distribution of magnetic particulates in a roadside snowpack based on magnetic, microstructural and mineralogical analyses. Geophysical Journal International.

[CR7] Chan LS, Yeung CH, Yim WWS, Or OL (1997). Magnetic susceptibility and distribution of heavy metals in contaminated sea-floor sediments of Hong Kong. Eng Geol Environ.

[CR8] Chaparro MAE, Krishnamoorthy N, Chaparro MAE, Lecomte KL, Mullainathan S, Mehra R, Sinito AM (2015). Magnetic, chemical and radionuclide studies of river sediments and their variation with different physiographic regions of Bharathapuzha River, southwestern India. Stud Geophys Geod.

[CR9] Chudaničová M, Hutchinson SM, Hradecký J, Sedláček J (2016). Environmental magnetism as a dating proxy for recent overbank sediments of (peri-)industrial regions in the Czech Republic and UK. Catena.

[CR10] Cowan EA, Gaspari DP, Brachfeld SA, Seramur KC (2015). Characterization of coal ash released in the TVA Kingston spill to facilitate detection of ash in river systems using magnetic methods. Fuel.

[CR11] Day R, Fuller M, Schmidt VA (1977). Hysteresis properties of titanomagnetites: grain size and compositional dependence. Phys Earth Planet Inter.

[CR12] Dearing JA, Hay KL, Baban SMJ, Huddleston AS, Wellington EMH, Loveland PJ (1996). Magnetic susceptibility of soil: an evaluation of conflicting theories using a national data set. Geophys J Int.

[CR13] Desenfant F, Petrovský E, Rochette P (2004). Magnetic signature of industrial pollution of stream sediments and correlation with heavy metals: case study from south France. Water Air Soil Pollut.

[CR14] Dunlop DJ (2002) Theory and application of the Day plot (Mrs/Ms versus Hcr/Hc): 2. Application to data for rocks, sediments, and soils. J Geophys Res 107(B3). 10.1029/2001JB000487

[CR15] Dunlop DJ, Özdemir Ö (1997). Rock magnetism: fundamentals and frontiers.

[CR16] Flanders PJ (1994). Collection, measurement, and analysis of airborne magnetic particulates from pollution in the environment. J Appl Phys.

[CR17] Frančišković-Bilinski S, Bilinski H, Maldini K, Milović S, Zhang Q, Appel E (2017). Chemical and magnetic tracing of coal slag pollutants in karstic river sediments. Environ Earth Sci.

[CR18] Georgeaud V, Rochette P, Ambrosi JP, Vandamme D, Williamson D (1997). Relationship between heavy metals and magnetic properties in a large polluted catchment: the Etand de Berre (South of France). Phys Chem Earth.

[CR19] Górka-Kostrubiec B (2015). The magnetic properties of indoor dust fractions as markers of air pollution inside buildings. Build Environ.

[CR20] Górka-Kostrubiec B, Król E, Jeleńska M (2012). Magnetic measurements of polluted filters in relation to meteorological conditions e case study from Warsaw. Stud Geophys Geod.

[CR21] Górka-Kostrubiec B, Jeleńska M, Król E (2014). Magnetic signature of indoor air pollution: household dust study. Acta Geophysica.

[CR22] Haus N, Zimmermann S, Wiegand J, Sures B (2007). Occurrence of platinum and additional traffic related heavy metals in sediments and biota. Chemosphere.

[CR23] Hjortenkrans DST, Bergbäck BG, Häggerud AV (2007). Metal emissions from brake linings and tires: case studies of Stockholm, Sweden 1995/1998 and 2005. Environ Sci Technol.

[CR24] Horng CS, Huh CA, Chen KH, Huang PR, Hsiung KH, Lin HL (2009). Air pollution history elucidated from anthropogenic spherules and their magnetic signatures in marine sediments offshore of southwestern Taiwan. J Marine Syst.

[CR25] Hu S, Wang Y, Appel E, Zhu Y, Hoffmann V, Shi C, Yu Y (2003). Magnetic responses to acidification in Lake Yangzonghai, SW China. Phys Chem Earth.

[CR26] Jordanova D, Hoffmann V, Thomas Fehr K (2004). Mineral magnetic characterization of anthropogenic magnetic phases in the Danube river sediments (Bulgarian part). Earth Planet Sci Lett.

[CR27] Jordanova D, Jordanova N, Hoffmann V (2006). Magnetic mineralogy and grain-size dependence of hysteresis parameters of single spherules from industrial waste products. Phys Earth Planet Inter.

[CR28] Jordanova D, Jordanova N, Lanos P, Petrov P, Tsacheva T (2012). Magnetism of outdoor and indoor settled dust and its utilization as a tool for revealing the effect of elevated particulate air pollution on cardiovascular mortality. Geochem Geophys Geosyst.

[CR29] Kim W, Doh SJ, Park YH, Yun ST (2007). Two-year magnetic monitoring in conjunction with geochemical and electron microscopic data of roadside dust in Seoul, Korea. Atmos Env.

[CR30] King JG, Williams W (2000). Low-temperature magnetic properties of magnetite. J Geophys Res.

[CR31] Krishnamoorthy N, Mullainathan S, Mehra R, Chaparro MAE (2014). Radiation impact assessment of naturally occurring radionuclides and magnetic mineral studies of Bharathapuzha river sediments, South India. Environ Earth Sci.

[CR32] Kukier U, Ishak CF, Sumner ME, Miller WP (2003). Composition and element solubility of magnetic and non-magnetic fly ash fractions. Environ Pollut.

[CR33] Kutchko BG, Kim AG (2006). Fly ash characterization by SEM–EDS. Fuel.

[CR34] Li F, Li G, Ji J (2011). Increasing magnetic susceptibility of the suspended particles in Yangtze River and possible contribution of fly ash. Catena.

[CR35] Magiera T, Jabłońska M, Strzyszcz Z, Rachwał M (2011). Morphological and mineralogical forms of technogenic magnetic particles in industrial dusts. Atmos Env.

[CR36] Maher BA, Moore C, Matzka J (2008). Spatial variation in vehicle-derived metal pollution identified by magnetic and elemental analysis of roadside tree leaves. Atmos Env.

[CR37] Morawski W (2011). Objaśnienia do Mapy Geologicznej Polski 1:200 000, ark.

[CR38] Morawski W, Pielach M (2011). Mapa Geologiczna Polski 1:200 000, ark.

[CR39] Moreno E, Sagnotti L, Dinares-Turell J, Winkler A, Cascella A (2003). Biomonitoring of traffic air pollution in Rome using magnetic properties of tree leaves. Atmos Env.

[CR40] Moskowitz BM (1991) Hitchhiker’s guide to magnetism. http://www.irm.umn.edu/hg2m/hg2m_d/hg2m_d.html)

[CR41] Muxworthy A, Matzka J, Petersen N (2001). Comparison of magnetic parameters of urban atmospheric particulate matter with pollution and meteorological data. Atmos Env.

[CR42] Nguyen TTH, Zhang WG, Li Z, Li J, Ge C, Liu JY, Bai XX, Feng H, Yu LZ (2016) Magnetic properties of sediments of the Red River, Vietnam: effect of sorting on the source-to-sink pathway and its implications for environmental reconstruction. Geochem Geophys Geosyst:172. 10.1002/2015GC006089

[CR43] O’Reilly W (1984). Rock and mineral magnetism.

[CR44] Petrovský E, Kapička A, Jordanova N, Knab M, Hoffmann V (2000). Low-field magnetic susceptibility: a proxy method of estimating increased pollution of different environmental systems. Environ Geol.

[CR45] Petrovský E, Zbořil R, Grygar TM, Kotlík B, Novák J, Kapička A, Grison H (2013). Magnetic particles in atmospheric particulate matter collected at sites with different level of air pollution. Studia Geophysica et Geodaetica.

[CR46] Prajith A, Rao VP, Kessarkar PM (2015). Magnetic properties of sediments in cores from the Mandovi estuary, western India: inferences on provenance and pollution. Mar Pollut Bull.

[CR47] Sarnacka Z (1980). Szczegółowa Mapa Geologiczna Polski 1:50 000. Arkusz Warszawa Wschód (524).

[CR48] Sarnacka Z (1980). Objaśnienia do Szczegółowej mapy geologicznej Polski 1:50 000. Arkusz Warszawa Wschód (524).

[CR49] Schauer JJ, Lough GC, Shafer MM, Christensen WF, Arndt MF, DeMinter JT, Park JS (2006). Characterization of metals emitted from motor vehicles. Res Rep Health Eff Inst.

[CR50] Scholger R (1998). Heavy metal pollution monitoring by magnetic susceptibility measurements applied to sediments of the river Mur (Styria, Austria). Eur J Environ Eng Geophys.

[CR51] Singh AK, Hasnain SI, Banerjee DK (2003). Grain size and geochemical portioning of heavy metals in sediments of the Danodar River e a tributary of the lower Ganga. India Environ Geol.

[CR52] Suresh G, Ramasamy V, Meenakshisundaram V, Venkatachalapathy R, Ponnusamy V (2011). Influence of mineralogical and heavy metal composition on natural radionuclide concentrations in the river sediments. Appl Radiat Isot.

[CR53] Szczepaniak-Wnuk I, Górka-Kostrubiec B (2018) Magnetic study of sediments from the Vistula River in Warsaw - preliminary results. Magnetometry in environmental sciences. GeoPlanet: Earth And Planetary Sciences. Springer Cham. 10.1007/978-3-319-60213-4_2

[CR54] Tauxe L (2005) Lectures in paleomagnetism. http://earthref.org/MAGIC/books/Tauxe/2005/

[CR55] Thompson R, Oldfield F (1986). Environmental magnetism.

[CR56] Tomassi-Morawiec H (2016). Atlas geochemiczny Warszawy i okolic.

[CR57] Tomlinson DC, Wilson JG, Harris CR, Jeffrey DW (1980). Problems in assessment of heavy metals in estuaries and the formation of a pollution index. Helgol Meeresunters.

[CR58] Ugwu I, Igbokwe O (2019) Sorption of heavy metals on clay minerals and oxides: a review. doi:10.5772/intechopen.80989

[CR59] Vagnetti R, Miana P, Fabris M, Pavonia B (2003). Self-purification ability of a resurgence stream. Chemosphere.

[CR60] Wang L, Hu S, Ma M, Wang X, Wang Q, Zhang Z, Shen J (2018). Responses of magnetic properties to heavy metal pollution recorded by lacustrine sediments from the Lugu Lake, Southwest China. Environ Sci Pollut Res.

[CR61] Zhang C, Qingqing Q, Piper JDA, Huang B (2011). Assessment of heavy metal pollution from a Fe-smelting plant in urban river sediments using environmental magnetic and geochemical methods. Environ Pollut.

